# β-1,3-Glucanase production as an anti-fungal enzyme by phylogenetically different strains of the genus *Clostridium* isolated from anoxic soil that underwent biological disinfestation

**DOI:** 10.1007/s00253-020-10626-8

**Published:** 2020-04-24

**Authors:** Atsuko Ueki, Toshiaki Takehara, Gen Ishioka, Nobuo Kaku, Katsuji Ueki

**Affiliations:** 1 Faculty of Agriculture, Yamagata University, 1-23, Wakaba-machi, Tsuruoka, Yamagata 997-8555 Japan; 2 NARO Western Region Agricultural Research Center, Hiroshima, 721-8514 Japan; 3 Present Address: NARO Technical Support Center of Central Region, Ibaraki, 305-8517 Japan

**Keywords:** Anaerobic bacteria, β-1,3-glucanase, Biocontrol of soil-borne pathogen, *Clostridium*, *Fusarium oxysporum* f. sp. *spinaciae*, Reductive soil disinfestation

## Abstract

Biological (or reductive) soil disinfestation (BSD or RSD) is a bioremediation process to control soil-borne plant pathogens using activities of indigenous bacteria in the soil. Three obligate anaerobic bacterial strains (TW1, TW10, and TB10), which were isolated from anoxic soil subjected to BSD treatments, were examined for their abilities to produce anti-fungal enzymes. All strains were affiliated with the different lineages of the genus *Clostridium*. The three strains decomposed β-1,3-glucans (curdlan and laminarin), and β-1,3-glucanase activities were detected from their culture supernatants with these glucans. The three strains also produced the enzyme with wheat bran as a growth substrate and killed the *Fusarium* pathogen (*Fusarium oxysporum* f. sp. *spinaciae*) in the anaerobic co-incubation conditions. Observation by fluorescence microscopy of the pathogen cells showed that the three strains had degraded the fungal cells in different manners upon co-incubation with wheat bran. When the three strains were cultivated with the dead cells or the cell wall samples prepared from the *Fusarium* pathogen, strain TW1 utilized these materials as easily decomposable substrates by releasing β-1,3-glucanase. When observed by fluorescence microscopy, it appeared that strain TW1 degraded the mycelial cell wall nearly thoroughly, with the septa remaining as undecomposed luminous rings. In contrast, the other two strains decomposed neither the dead cells nor the cell wall samples directly. The results indicate that the various anaerobic bacteria proliferated in the soil under the BSD treatments should play key roles as an organized bacterial community to eliminate fungal pathogens, namely by release of anti-fungal enzymes with different properties.

**Table:** 

**Key points** ***•****Three clostridial strains isolated from BSD-treated soils produced β-1,3-glucanase.* ***•****All strains killed the Fusarium pathogen in the anaerobic co-incubation conditions.* ***•****One of the strains produced β-1,3-glucanase with the fungal cell wall as a substrate.* ***•****The strain degraded the cell wall almost completely, except for the mycelial septa.*

**Electronic supplementary material:**

The online version of this article (10.1007/s00253-020-10626-8) contains supplementary material, which is available to authorized users.

## Introduction

Biological soil disinfestation (BSD) is a method to control or eliminate soil-borne plant pathogens before crop cultivation, using both biological materials and microbial activities in soil without the application of agricultural chemicals. BSD is also referred to as reductive or anaerobic soil disinfestation (RSD or ASD), as the development of reductive or anaerobic conditions of the soil during treatment is essential for successful elimination of the pathogens (Blok et al. 2000; Goud et al. 2004; Momma 2008; Momma et al. 2013; Strauss and Kluepfel 2015). The effectiveness of the method in suppressing soil-borne plant pathogens has been reported for various crops and plant diseases in different regions of the world (Browne et al. 2018; Butler et al. 2014; Huang et al. 2016; Mazzola et al. 2018; Meng et al. 2018; Messiha et al. 2007; Muramoto et al. 2014; Serrano-Pérez et al. 2017; Shennan et al. 2018; Shrestha et al. 2018). In this method, organic matter (usually plant biomass) needs to first be incorporated into the soil of the fields with irrigation water for moisture saturation. The soil surface is covered tightly by plastic sheets to maintain the anoxic soil conditions and the proper soil temperature for the treatment. Soil-borne plant pathogens that are present in the soil should be killed or inactivated under the anoxic conditions maintained for ~ 3 weeks. Soon after removal of the covering sheets and ploughing, the soil should be suitable for crop cultivation. The key point of this method is to maintain the highly reductive conditions of the soil in the presence of the organic matter that is incorporated.

Soil conditions of BSD treatments should be drastically changed with the following major processes: the decomposition of incorporated organic matter by various indigenous microbes that accompanies the elimination of O_2_ and the development of reductive or anoxic conditions; the production of fermentation end products (mainly fatty acids) and toxic compounds, such as indole, skatole, and H_2_S, by various microbes; and changes in pH (Macfarlane and Macfarlane 1995; Ueki et al. 2018). These changes may comprehensively affect plant pathogens in soil and cause their suppression or elimination from the soil.

In our previous studies, we reported that BSD treatments that use different types of biomass, such as wheat bran or *Brassica juncea* plants, clearly decreased the pathogen populations (*Fusarium oxysporum* f. sp. *lycopersici* [wilt pathogen of tomato] and *F. oxysporum* f. sp. *spinaciae* [wilt pathogen of spinach]) that were incorporated into soil in model experiments (Mowlick et al. 2012, 2013a). Furthermore, BSD treatments conducted in different field conditions also suppressed incidences of wilt disease of spinach cultivation (Mowlick et al. 2013b, c, 2014). We analyzed bacterial compositions in these soil samples subjected to the BSD treatments, using the clone library method. In these soil samples, obligate anaerobic bacterial groups in the class *Clostridia* were found to be exclusively and collectively dominant with some other aerobic or facultative anaerobic bacteria. We isolated anaerobic bacteria from the soil samples subjected to the treatments in the model experiments shown above (Mowlick et al. 2012, 2013a) to investigate their role in suppressing soil-borne plant pathogens. In the previous studies (Ueki et al. 2017, 2019), we explored the degradation of the fungal cell wall of ascomycetes using the two anaerobic bacterial strains (H110 and TB8 identified as *Clostridium beijerinckii*) out of these isolates. The two anaerobic bacterial strains that produced β-1,3-glucanase and chitosanase were shown to almost completely decompose cells of the ascomycete fungal pathogen, *F. oxysporum* f. sp. *spinaciae*, in anaerobic co-incubation conditions. In the present study, we further investigated the abilities of other anaerobic strains (strains TW1, TW10, and TB10) that were isolated from the BSD-treated soil samples to suppress the *Fusarium* pathogens. Based on the 16S rRNA gene sequences, the three strains were affiliated with phylogenetically different lineages of the genus *Clostridium* from one another. The three strains were similar in utilizing β-1,3-glucans (curdlan and laminarin) as growth substrates and producing β-1,3-glucanase in the culture supernatants, whereas none of them decomposed chitosan. Thus, the three strains had different physiological properties compared to strains H110 and TB8 (Ueki et al. 2017, 2019) for the decomposition of polysaccharides that comprise the cell wall of the ascomycetes. It was also shown that although the three clostridial strains are similar in producing β-1,3-glucanase as an anti-fungal enzyme, the strains attack the cells of the *Fusarium* pathogen of spinach wilt disease in different manners.

## Materials and methods

### Isolation and cultivation of anaerobic bacteria

The anaerobic bacterial strains were isolated from the soil samples subjected to the BSD treatments in the model experiments incorporated with wheat bran or *Brassica juncea* plants (treated at 30 °C for 17–18 days) conducted in our previous studies in Japan at the Tokushima Agricultural Research Center (Mowlick et al. 2012) or at the NARO Western Region Agricultural Research Center, Hiroshima (Mowlick et al. 2013a). The strains were named with TW (wheat bran-incorporated) or TB (*Brassica*-incorporated) for the isolates from Tokushima, and an initial letter H (wheat bran-incorporated) and E (*Brassica*-incorporated) for those from Hiroshima, respectively. The purity of the strains was confirmed through repetition of the anaerobic roll tube method for each isolate (Holdeman et al. 1977) as well as observation of cell morphology by microscopy. The strains were cultivated under anaerobic conditions at 30 °C using a peptone-yeast extract (PY) medium as a basal medium with oxygen-free mixed gas (95% N_2_ and 5% CO_2_) in the headspace as described earlier (Ueki et al. 2017, 2019). Each test tube containing the medium was closed tightly with an inner butyl rubber stopper equipped with an outer screw cap. The PY broth contained (per liter) 10 g trypticase (BD BBL, Sparks, USA), 5 g yeast extract, 0.2 g Na_2_CO_3_, 0.3 g l-cysteine·HCl·H_2_O (a reducing agent), and 1 mg sodium resazurin (a redox indicator), as well as salt solutions (Satoh et al. 2002). The PY medium supplemented with (per liter) 0.25 g of each glucose, cellobiose, maltose, and soluble starch, as well as 15 g agar, designated as PY4S medium, was used for isolation and maintenance of strains as agar slant cultures. Before autoclaving, the pH of all the media was adjusted to 7.2–7.4 with NaOH solution. PY broth medium supplemented with glucose at 10 g/l was designated as PYG broth and was used for cultivation of the strains as inoculums for various cultural conditions. Inoculation of pre-cultivated cells to the media was carried out under the stream of oxygen-free mixed gas (95% N_2_ and 5% CO_2_).

### Phylogeny of isolates based on 16S rRNA gene sequences and construction of the phylogenetic tree

Genomic DNA of each strain was extracted from cell biomass cultivated in PYG broth. Almost full-length of 16S rRNA genes were amplified using a primer pair B27f (5′-AGA GTT TGA TYM TGG CTC AG-3′) and U1492r (5′-GGY TAC CTT GTT ACG ACT T-3′). The composition of the reaction mixture and PCR amplification conditions were described by Mowlick et al. (2012). Sequence analysis was conducted using sequencing primers B27f and U1492R as well as U515f (5′-GTG YCA GCM GCC GCG GTAA-3′) (Mowlick et al. 2013a) by the dye terminator method at the Premix Sequencing Service of Takara Bio Inc. (Kusatsu, Japan).

### Phenotypic properties of anaerobic strains

Cell morphology and spore formation were examined by microscopy. Motility of the cells was evaluated by phase contrast microscopy. Physiological examination of the isolates was performed as reported previously (Ueki et al. 2016, 2017, 2019). All the cultivation experiments were performed at least in duplicate, and reproducibility of the results was confirmed. Growth of the bacterial strains in the presence of various carbohydrates under the anaerobic conditions was examined by using PY broth as a basal medium supplemented with 0.5 or 1% (*w*/*v*) of respective substrate or by API 20 Microbial Identification Kit (bioMérieux, Lyon, France). Curdlan (Wako Ind. Ltd., Osaka, Japan), laminarin (Nacalai Tesque, Kyoto, Japan), glucan from black yeast (yeast glucan) (Tokyo Chemical Industry Co. Ltd., Tokyo, Japan), chitin (from crab shells, Wako Ind. Ltd., Osaka, Japan), and chitosan (deacetylated chitin from crab shells, Wako Ind. Ltd., Osaka, Japan) were used as polysaccharide substitutes to the components of the ascomycetes fungal cell wall. Curdlan is a linear polymer of β-1,3-glucan and laminarin is composed of β-1,3-glucan and β-1,6-glucan (9.51:1 for laminarin from *Laminaria digitata*) (Liu et al. 2018). Chitin and chitosan are polymers of *N*-acetylglucosamine (GlcNAc) and glucosamine, respectively. The composition of the commercially available reagent “yeast glucan” used in this study has not been presented. Curdlan, yeast glucan, chitin, and chitosan were insoluble in water, whereas laminarin was soluble. In autoclaved PY broth supplemented with insoluble substrates (0.5%, *w*/*v*), curdlan precipitated as a lumpy material of hydrogel, yeast glucan diffused as cloudy precipitates, and chitin as well as chitosan produced a translucent hydrogel. The insoluble substrates were used without any further treatment. Wheat bran and dried leaves of *B. juncea* were also used as substrates for the cultivation of the strains to examine the production of the enzymes. Dried leaves of *B. juncea* were ground into coarse powder using a mortar and pestle for the ease of medium preparation. Wheat bran and *Brassica* leaves were added to PY broth (1%, *w*/*v*) and then the media were autoclaved, in which these substrates precipitated as solid sediment. All the cultivation experiments of the strains were carried out at 30 °C.

The substrate utilization abilities for each strain were determined by monitoring the increase in culture turbidity (OD_660_) as well as the quantity of the products in the culture supernatants as compared to those of the cultures cultivated without substrates (PY broth). Especially, the abilities to degrade insoluble substrates were determined by measuring fermentation products in the culture supernatants after the cultivation. Fermentation products, such as volatile fatty acids (VFAs), alcohols, and gases (H_2_ and CO_2_), were analyzed by gas chromatography (GC) (Hitachi G-3000, G-5000 or G-163 Tokyo, Japan) as described previously (Ueki et al. 2017, 2019). All the experiments for measurement of the products were performed in duplicates.

### Determination of β-1,3-glucanase activity

β-1,3-Glucanase activity was determined using supernatants of cultures supplemented with different substrates as described previously (Ueki et al. 2019). After cultivation in PY broth containing respective substrate as shown below, the culture supernatants were collected by centrifugation at 25,000×*g* for 20 min and were stored at − 20 °C until use. β-1,3-Glucanase activity was assessed with laminarin (from *L. digitata*, Sigma-Aldrich, St. Louis, USA) as a water-soluble substrate according to the standard method (Aktuganov et al. 2007; Zacky and Ting 2013). The reaction mixture was composed of 1 ml of culture supernatant as an enzyme solution, 0.5 ml of laminarin solution (0.5%, *w*/*v*), and 1 ml of 10 mM potassium phosphate buffer (pH 7.0). The reaction mixture was incubated for 15 and 30 min at 38 °C in a water bath and was stopped by transferring in ice-cold water. The concentration of reducing sugar in the reaction mixture was measured by 3,5-dinitrosalicylic acid (DNS) method (Miller 1959) at 535 nm based on the standard curve prepared by using different concentrations of glucose. One unit of enzyme activity was defined as the amount of enzyme catalyzing the formation of 1 μmol reducing sugar (as glucose equivalent) in 60 min under conditions described above. The enzyme assay was performed at least in four replicates.

### Effects of the growth of the bacterial strains on the survival of the *Fusarium* pathogen under anaerobic conditions

Effects of growth of the anaerobic bacterial strains on the survival of the fungal pathogen were examined by anaerobic co-incubation of each strain with the pathogen in PY broth containing wheat bran as a growth substrate. The nitrate-non-utilizing (*nit*) mutant of *F. oxysporum* f. sp. *spinaciae* strain M2-1 (= MAFF150001) (Mowlick et al. 2013a; Takehara et al. 2003; Ueki et al. 2017, 2019) was used as the wilt pathogen of spinach. Strain M2-1 was streaked on potato dextrose agar (PDA) plates and aerobically cultivated for 7 days at 28 °C. The PDA plates with densely overgrown mycelia were cut into square pieces (approximately 6 mm × 6 mm). Five such agar pieces were inoculated into PY broth (10 ml in each test tube) containing wheat bran under the stream of oxygen-free gas (95% N_2_ and 5% CO_2_). Cells of each bacterial strain (0.1 ml of 24 h old culture cultivated in PYG broth) were inoculated into the PY broth containing both the agar pieces and wheat bran in the test tubes (20–25 replicates for each anaerobic bacterial strain) and were incubated anaerobically at 30 °C. Simultaneously, replicates of the control culture without inoculation of the anaerobic bacterial strains (i.e., the *Fusarium* pathogen alone in PY broth with wheat bran) were also incubated under the anaerobic conditions. Agar pieces were destructively taken out from four or five culture tubes randomly on each sampling day (7, 14, and 21 days) during the incubation period and were cut into smaller pieces (approximately 2 mm × 6 mm), which were immediately placed sideways onto two fresh PDA plates (10 pieces in total). The agar plates were incubated at 28 °C under aerobic conditions to examine mycelial growth of the pathogen from each agar piece. Based on the microscopic examination of the mycelial cells on the agar pieces, more than 1 × 10^4^ cells were present on each agar piece placed on the PDA plates (Ueki et al. 2017). The remaining agar sections taken out from the PY broth were stored in 5% (*v*/*v*) formaldehyde solution to observe the fungal cells on the PDA sections by fluorescence microscopy.

### Preparation of whole cell dead biomass and crude cell wall samples of *F. oxysporum* f. sp. *spinaciae* as substrates for cultivation of the anaerobic strains

Freeze-dried whole-cell dead biomass and crude cell wall samples of *F. oxysporum* f. sp. *spinaciae* were prepared by the same procedure as described earlier (Ueki et al. 2017, 2019). The *Fusarium* pathogen strain M2-1 was cultivated in the potato dextrose (PD) broth for 7 days at 28 °C by using a reciprocal shaker (100 rpm). The fungal biomass was harvested by centrifugation at 10,000×*g* for 20 min, which was then resuspended in distilled water and washed again by centrifugation at 10,000×*g* for 20 min. The resulting pellet was freeze-dried and stored at 4 °C. The freeze-dried mycelial cells were added to PY broth (1%, *w*/*v*), which was then autoclaved to kill the pathogen. The dead cell biomass precipitated as dark purple solids in the medium after autoclaving.

The mycelium of strain M2-1 for preparation of the cell wall sample was cultivated and harvested as shown above. The cell biomass collected by centrifugation was resuspended in distilled water and disrupted using ultra-sonication (Branson sonifier 250, Branson Ultrasonics, Ltd. Danbury, USA) until disruption of almost all cells was confirmed by microscopic observation. The cell wall sample was washed four times with distilled water by centrifugation at 10,000×*g* for 20 min. The pellet was resuspended in a 1% (*w*/*v*) solution of sodium dodecyl sulfate (SDS) and shaken vigorously. The crude cell wall was collected and washed three times with centrifugation in distilled water. The isolated cell wall preparation was freeze-dried and kept at 4 °C until use. Decomposition of the cell wall preparation by the anaerobic strains was analyzed by adding the freeze-dried sample into PY broth at 0.5% (*w*/*v*) in a similar way as that of the carbohydrate utilization test. The cell wall preparation precipitated as white particles in the PY broth.

### Observation of cells of *F. oxysporum* f. sp. *spinaciae* by fluorescence microscopy

After the anaerobic incubation of the mycelial cells of *F. oxysporum* f. sp. *spinaciae* strain M2-1 (live cells on PDA sections, freeze-dried dead whole cells or cell wall preparation), the incubated mycelial cells were harvested from the medium and stored in 5% (*v*/v) formaldehyde solution. For fluorescence microscopy, calcofluor white stain (Sigma-Aldrich Co. LLC, St. Louis, USA) was used to observe the mycelial samples according to the manufacturer’s instructions. A fluorescence microscope (Olympus BX51, Olympus Corp. Tokyo, Japan) equipped with Million Pixel Camera System (Carl Zeiss, Oberkochen, Germany) was used.

### Data analysis

Multiple alignments of the sequences with reference sequences from the GenBank database were performed using the BLAST software (Altschul et al. 1997). A phylogenetic tree was constructed with the neighbor-joining method (Saitou and Nei 1987) by using the CLUSTAL W package (Thompson et al. 1994). All gaps and unidentified base positions in the alignments were excluded before sequence assembly.

### Accession numbers of the sequences and strain numbers deposited in the culture collection

Nucleotide sequences of 16S rRNA gene of the strains isolated from the BSD-treated soil samples were deposited in DDBJ/GenBank under the accession numbers LC020489–LC020515. The numbers of the three strains mainly used in this study are LC020506 (TW1), LC020495 (TW10), and LC020496 (TB10). The strains isolated from the BSD soil samples were deposited in NBRC (NITE Biological Resource Center, Kisarazu, Japan) as NBRC112093–1120102. The numbers of the strains mainly used in this study are NBRC1102097 (= TW1), NBRC1102099 (= TW10), and NBRC112095 (= TB10).

## Results

### Phylogeny of anaerobic bacterial strains isolated from soil samples subjected to BSD treatments

**Fig. 1:**
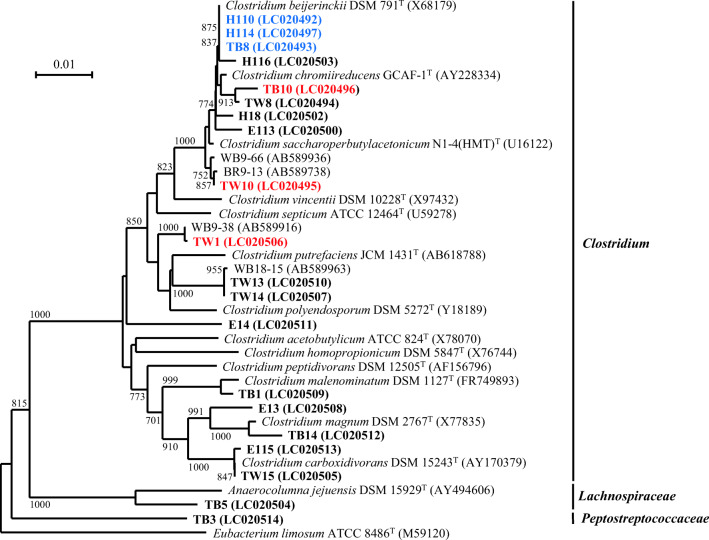
Neighbor-joining tree based on 16S rRNA gene sequences showing the phylogenetic relationship of the strains and the clones isolated from the soil samples subjected to BSD treatments to related members in the order *Clostridiales* in the class *Clostridia*. Strains and clones: red bold letters, strains producing β-1,3-glucanase and examined in this study; blue bold, strains producing both β-1,3-glucanase and chitosanase (Ueki et al. 2017, 2019); black bold, others; black normal letters, clones. The strains were named with TW (wheat bran-incorporated) or TB (*Brassica*-incorporated) for the isolates from Tokushima, and an initial letter H (wheat bran-incorporated) and E (*Brassica*-incorporated) from Hiroshima, respectively. The name of each clone starts with the soil sample designation: WB (wheat bran-incorporated) or BR (*Brassica*-incorporated) soil in Tokushima; H1, wheat bran-incorporated soil in Hiroshima. Accession numbers of the sequences are shown in the parentheses. The sequence length compared is 819 bp. Bootstrap values (expressed as percentages of 1000 replications) above 70% are shown at branch nodes. The 16S rRNA gene sequence of *Eubacterium limosum* ATCC 8486^T^ was used as the outgroup. Bar, 1% estimated difference in nucleotide sequence

Approximately 60 strains were finally purified from the 4 BSD-treated soil samples, and certain basic phenotypic properties were examined for all isolates. All isolates were obligate anaerobes and most of their cells were motile, spore-forming, and Gram-stain-positive rods. Most of the isolates produced acetate and butyrate, and some strains produced ethanol and *n*-butanol as fermentation end products in the PYG broth. Based on the obtained properties, 20 strains were selected as representatives for further phylogenetic analysis based on 16S rRNA gene sequences. All the selected 20 strains were affiliated with the class *Clostridia* within the phylum *Firmicutes*, and most of the strains, except for the two strains (TB3 and TB5), were classified in the genus *Clostridium* (Lawson and Rainey 2016; Rainey et al. 2009) (Fig. 1). Nine strains (from strain H110 at the top of the tree to strain TW10) were assigned to the same clade with *Clostridium beijerinckii*, *Clostridium chromiireducens*, and *Clostridium saccharoperobutylacetonicum*. Other strains were assigned to several other groups or lineages with the respective closely related *Clostridium* species.

### Ability of the anaerobic strains to decompose polysaccharides related to ascomycetes fungal cell wall

Nineteen strains (except strain TB3) out of 20 strains actively grew in the presence of glucose in PY broth (PYG medium) under the anaerobic conditions, which demonstrated the saccharolytic properties of these strains. Most strains produced acetate, butyrate, and gases (H_2_ and CO_2_) in PYG or PY broth. To understand the potential of the strains to decompose the fungal cell wall of ascomycetes, the ability of the strains to decompose major polysaccharides comprising the ascomycetes fungal cell wall (curdlan as β-1,3-glucan, chitin, and chitosan) was examined (Table 1). Three strains (H110, H114, and TB8) decomposed both curdlan and chitosan and produced *n*-butanol and ethanol in addition to acetate, butyrate, and gases. Three strains (TW1, TW10, and TB10) decomposed curdlan, although not chitosan, and produced acetate and butyrate, and gases. None of the remaining 14 strains decomposed β-1,3-glucan or chitosan. None of the strains decomposed chitin. The strains which decomposed both β-1,3-glucan and chitosan were closely related to one another in the genus *Clostridium*, whereas the three curdlan (β-1,3-glucan) decomposers were dispersed in phylogenetically different lineages within the genus *Clostridium* (Fig. 1). Strains H110 and TB8 were identified as *C. beijerinckii*; further, their degradation of the fungal cell wall and production of β-1,3-glucanase and chitosanase have been previously reported (Ueki et al. 2017, 2019). In this study, the three β-1,3-glucan decomposers (TW1, TW10, and TB10) were further investigated for their potential to degrade fungal cells and to suppress soil-borne fungal plant pathogens under the BSD treatment conditions.

**Table 1:** Grouping of representative clostridial isolates from BSD-treated soil samples based on their physiological properties

Group	Strain	Products	Utilization of substrate				Reference
			Glucose	Curdlan	Chitosan	Chitin	
1	H110, H114, TB8	Acetate, butyrate, ethanol, *n*-butanol, H_2_, CO_2_	+	+	+	−	Ueki et al. (2017, 2019)
2	TW1, TW10, TB10	Acetate, butyrate, H_2_, CO_2_	+	+	−	−	This study
3	H18, H116, TW8, TW13, TW14, TW15, E13, E14, E113, E115, TB1	Acetate, butyrate, H_2_, CO_2_	+	−	−	−	
4	TB5, TB14	Acetate, ethanol, H_2_, CO_2_	+	−	−	−	
5	TB3	Acetate, H_2_, CO_2_	−	−	−	−	

All strains are obligately anaerobic, Gram-stain positive rods. +, positive; −, negative

### Phylogeny and characteristics of strains TW1, TW10, and TB10

The three strains were assigned to phylogenetically different lineages in the genus *Clostridium*, as shown above (Fig. 1). Cell morphologies of the three strains were distinct from one another (Supplemental Fig. S1). The closest relatives to strains TW1, TW10, and TB10 were *Clostridium polyendosporum* (96.1% sequence similarity of the 16S rRNA gene), *C. saccharoperbutylacetonicum* (99.1%), and *C. chromiireducens* (99.2%) (Supplemental Table S1). Sequence similarities between strain TW1 and the other two strains were 91% for strain TW10 and 92% for strain TB10, and the sequence similarity between strains TW10 and TB10 was 94%. Carbohydrate utilization and other characteristics of the three strains are shown in Supplemental Table S2. All three strains produced acids from various carbohydrates. Further, all strains were negative for the hydrolysis of aesculin and urea and the production of indole.

GlcNAc and glucosamine are monomers composed of chitin and chitosan, respectively. The three strains were cultivated with these substrates in PY broth. Fermentation end products of the strains from these substrates as well as from glucose are shown in Table 2. All strains produced minor amounts of acetate, butyrate, H_2_, and CO_2_ from the PY broth that lacked supplemented substrates. All strains showed good growth in the presence of glucose, and produced substantial amounts of acetate, butyrate, and gases. A higher concentration of acetate was detected for strain TW1, as compared to strains TW10 and TB10. Both GlcNAc and glucosamine also supported growth and formation of products of the three strains. All strains produced much higher amounts of acetate from GlcNAc, as compared to from glucose or glucosamine, which suggests that acetate formation occurs through the deacetylation of GlcNAc for all strains.

**Table 2:** End products of strains TW1, TW10, and TB10 from monosaccharides constituting ascomycete fungal cell wall polysaccharides

Strain	Substrate	Products (mmol/l)			
		Acetate	Butyrate	H_2_	CO_2_
TW1	None (PY)	3.13 ± 0.35	1.62 ± 0.08	7.41 ± 0.08	8.78 ± 0.19
	Glucose	12.4 ± 0.40	16.1 ± 0.60	41.7 ± 1.57	39.4 ± 1.04
	GlcNAc	29.3 ± 1.12	12.5 ± 0.61	29.7 ± 2.42	29.0 ± 1.05
	Glucosamine	6.10 ± 0.28	17.2 ± 0.63	11.0 ± 1.56	0.70 ± 0.10
TW10	None (PY)	0.85 ± 0.01	1.76 ± 0.12	5.18 ± 0.61	7.58 ± 0.60
	Glucose	7.20 ± 0.30	13.2 ± 0.55	24.2 ± 0.10	20.5 ± 0.32
	GlcNAc	22.9 ± 0.28	14.4 ± 0.36	19.2 ± 0.88	22.4 ± 0.25
	Glucosamine	5.40 ± 0.40	13.5 ± 0.34	9.50 ± 0.03	0.60 ± 0.05
TB10	None (PY)	1.95 ± 0.17	2.15 ± 0.14	7.50 ± 0.10	8.68 ± 0.08
	Glucose	3.84 ± 0.20	13.9 ± 0.16	21.4 ± 0.09	20.4 ± 0.05
	GlcNAc	17.2 ± 1.02	17.9 ± 0.34	21.5 ± 1.09	25.5 ± 1.38
	Glucosamine	3.82 ± 0.12	25.4 ± 1.27	10.9 ± 1.30	0.70 ± 0.06

Concentrations of substrates, 1.0% (*w*/*v*); GlcNAc, *N*-acetylglucosamine

Cultivated for 7 days for all substrates. All data are mean values (± SD) of duplicate experiments

### Growth of the three strains with different types of glucan and production of β-1,3-glucanase

Decomposition of different types of glucan (curdlan, laminarin, and yeast glucan) by the three strains was examined to determine the ability of decomposing polysaccharides related to the ascomycetes cell wall. Because curdlan and yeast glucan are insoluble substrates, determination of product amounts in the culture supernatants was essential to confirming their growth with these substrates (Table 3). Acetate and butyrate, which were comparable to the amounts from glucose (Table 2), were produced from curdlan by the three strains, suggesting that the three strains utilized β-1,3-glucan, whereby it served as a favorable growth substrate. Substantial amounts of acetate and butyrate were also produced from laminarin for all three strains. Acetate and butyrate were also produced from yeast glucan for all strains; however, the amounts of products were lower than those from curdlan or laminarin for all strains. Strain TW10 especially produced low amounts of products from yeast glucan.

**Table 3:** End products and β-1,3-glucanase activity of strains TW1, TW10, and TB10 in the culture supernatants grown with different types of glucan

Strain	Substrate	Products (mmol/l)		β-1,3-Glucanase (unit/ml)
		Acetate	Butyrate	
TW1	None (PY)	2.83 ± 0.01	2.11 ± 0.06	0.61 ± 0.24
	Curdlan	6.93 ± 0.02	9.01 ± 0.21	5.12 ± 0.95
	Laminarin	12.4 ± 0.17	15.6 ± 0.27	2.67 ± 0.23
	Yeast glucan	4.46 ± 0.15	6.32 ± 0.21	2.20 ± 0.09
TW10	None (PY)	1.23 ± 0.02	2.09 ± 0.04	0.15 ± 0.05
	Curdlan	3.85 ± 0.02	15.6 ± 0.12	3.77 ± 1.44
	Laminarin	8.86 ± 1.46	14.8 ± 0.12	1.37 ± 0.23
	Yeast glucan	1.35 ± 0.06	3.46 ± 0.21	0.76 ± 0.24
TB10	None (PY)	1.71 ± 0.13	2.98 ± 0.04	0.58 ± 0.05
	Curdlan	3.46 ± 0.50	15.8 ± 0.11	4.04 ± 0.27
	Laminarin	4.85 ± 0.26	10.0 ± 0.10	5.71 ± 0.04
	Yeast glucan	1.13 ± 0.02	6.47 ± 0.13	1.75 ± 0.13

Concentrations of substrates, 0.5% (*w*/*v*). Cultivated for 7 days for all substrates

Yeast glucan, glucan from black yeast (Tokyo Chemical Industry Co., Ltd)

Products, mean ± SD (*n* = 2); β-1,3-glucanase, mean ± SD (*n* = 4)

β-1,3-Glucanase activities of the strains were determined in the culture supernatants using these glucans. Low β-1,3-glucanase activities were detected in the supernatants of the PY broth for all strains. Although the activity levels differed depending on the strains and types of glucans used for cultivation, β-1,3-glucanase activity levels that were higher than those from the PY broth were detected for all strains in the supernatants cultivated with these substrates. Curdlan supported the production of high β-1,3-glucanase activity levels for all strains. Although the amounts of products of strain TB10 from laminarin were lower than those of the other two strains, a higher β-1,3-glucanase activity was detected. For strain TW10, activity detected in the culture with yeast glucan was low in accordance with the low product amounts. The enzyme activities in the culture supernatants that were cultivated with glucose (PYG) were as low as those in PY broth for all strains (data not shown).

### Enzyme activities of the anaerobic strains in the culture supernatants with wheat bran and *Brassica* leaves as substrates for cultivation

Wheat bran and *Brassica* plants are both popular organic materials that are incorporated into soil for BSD treatments. To confirm the production of β-1,3-glucanase of the three strains using these materials as growth substrates, the three strains were cultivated in the presence of wheat bran or *Brassica* leaves in PY broth for 3 weeks, and enzyme activities in the culture supernatants were determined in addition to the products (Table 4). Although the product amounts for strain TW1 were slightly low compared to when glucose was used as a substrate, all strains produced similar amounts of acetate and butyrate from wheat bran as they had from glucose (Table 3). This indicates that the wheat bran was a suitable substrate for growth for all the three strains. In accordance with the substantial amounts of products, β-1,3-glucanase activity was detected in the culture supernatants of all strains. A higher activity was detected in the culture of strain TW1 compared to the other two strains.

**Table 4:** End products and β-1,3-glucanase activity of strains TW1, TW10, and TB10 in the culture supernatants grown with wheat bran or *Brassica* leaves in PY broth

Strain	Substrate	Products (mmol/l)		β-1,3-Glucanase
		Acetate	Butyrate	(unit/ml)
TW1	Wheat bran	8.76 ± 0.15	9.37 ± 0.57	2.33 ± 0.11
	*Brassica* leaf	5.32 ± 0.04	4.81 ± 0.12	1.91 ± 0.04
TW10	Wheat bran	5.50 ± 0.82	13.9 ± 0.17	1.23 ± 0.16
	*Brassica* leaf	3.28 ± 0.82	5.23 ± 0.23	–
TB10	Wheat bran	3.04 ± 0.36	15.1 ± 0.15	1.07 ± 0.14
	*Brassica* leaf	3.41 ± 0.81	8.43 ± 0.08	–

Concentrations of substrates, 1% (*w*/*v*). Cultivated for 21 days

Products, mean ± SD (*n* = 2); β-1,3-glucanase, mean ± SD (*n* = 4)

– not detected

All strains produced lower amounts of acetate and butyrate from the *Brassica* plant biomass, as compared to wheat bran. For strain TW1, β-1,3-glucanase was detected, though the activity was lower than that of wheat bran. No activity was detected for the other two strains (Table 4).

### Time course study of β-1,3-glucanase activity levels during the anaerobic co-incubation of *F. oxysporum* f. sp. *spinaciae* strain M2-1 live cells with the anaerobic bacterial strains using wheat bran as a substrate in PY broth

**Fig. 2:**
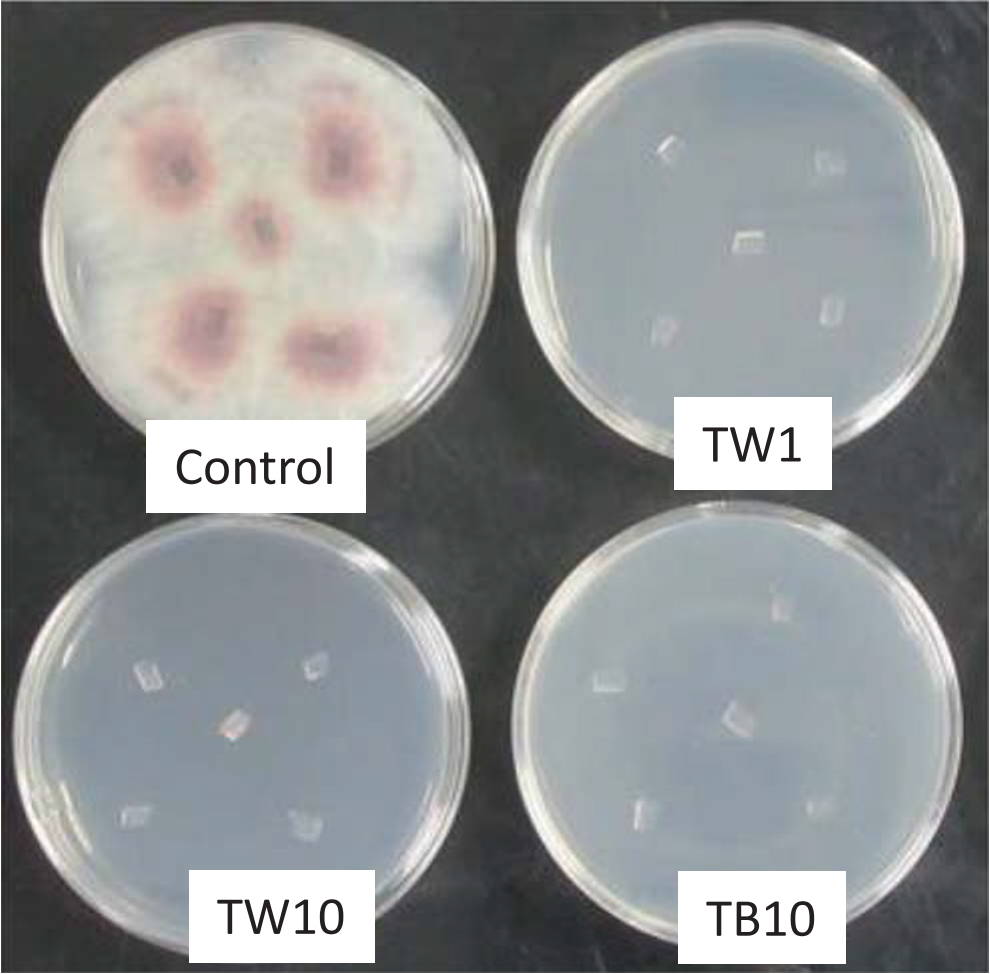
Growth of the mycelium of *Fusarium oxysporum* f. sp. *spinaciae* strain M2-1 from agar plate sections anaerobically incubated alone (control) or anaerobically co-incubated with wheat bran (1% *w*/*v*) in PY broth for 7 days with strains TW1, TW10, or TB10 at 30 °C. Agar sections recovered from the anaerobic incubation were cut into smaller pieces (6 mm × 2 mm), and the 10 pieces were placed on the 2 fresh PDA plates (5 pieces per plate) on their sides and incubated at 28 °C under the aerobic conditions. The photo was taken 6 days after placement of the agar sections on the fresh PDA plates. The same results were obtained from the remaining PDA plates for all strains and the control

Wheat bran was found to support both growth and β-1,3-glucanase production of the three strains. Thus, live cells of *F. oxysporum* f. sp. *spinaciae* strain M2-1 grown on PDA sections were anaerobically co-incubated with each of the three strains for 3 weeks in PY broth containing wheat bran. Figure 2 shows the PDA plates with agar pieces recovered from the PY broth after 7 days of the anaerobic co-incubation and then incubated under aerobic conditions for 6 days after transfer onto the fresh PDA plates. The mycelium of the pathogen that was anaerobically incubated alone in PY broth containing wheat bran (control) grew normally under the aerobic conditions from all of 10 agar pieces placed on the 2 PDA plates, whereas none of the agar pieces that were co-incubated with each of the three anaerobic strains showed extension of the mycelium, thus indicating that the anaerobes inactivated all pathogen cells on the PDA pieces under the anaerobic co-incubation conditions for 7 days. The pathogen also grew without delay when the agar pieces of the control culture, which were anaerobically incubated alone in PY broth with wheat bran for 3 weeks, were placed on the PDA plates (data not shown), which suggests that the anaerobic incubation in PY broth with wheat bran did not result in distinct effects of the pathogen viability on the PDA pieces.

**Fig. 3:**
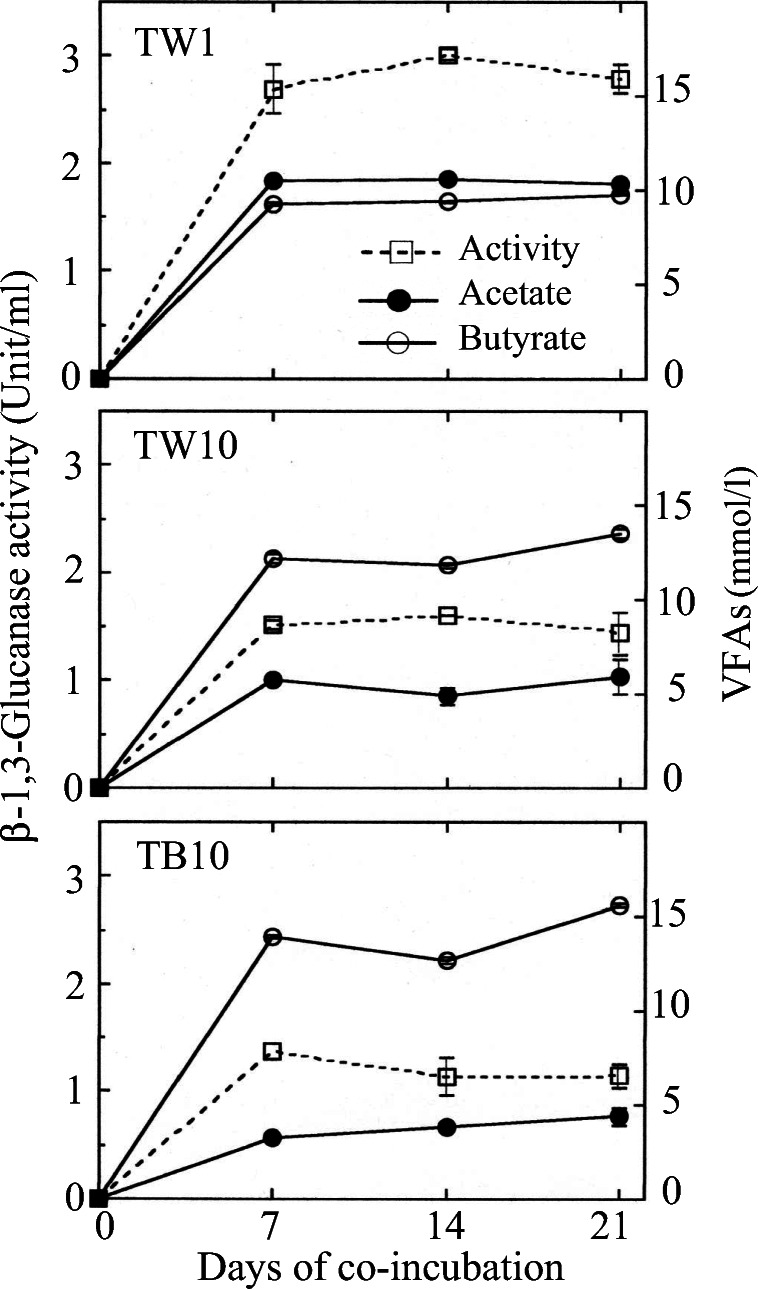
Time courses of β-1,3-glucanase activities and production of volatile fatty acids (VFAs) of strains TW1, TW10, and TB10 anaerobically co-incubated at 30 °C with *Fusarium oxysporum* f. sp. *spinaciae* strain M2-1 under the presence of wheat bran (1%, *w*/*v*) as a substrate in PY broth. VFAs were determined in duplicate cultures, and the enzyme activity was determined in four replicates. Symbols: □, β-1,3-glucanase; ●, acetate; ○, butyrate. Error bars represent the standard deviations

Changes in the product concentrations and β-1,3-glucanase activities of the co-incubations are shown in Fig. 3. Strain TW1 produced similar amounts of acetate and butyrate until day 7 of the co-incubation, and the concentrations were maintained for 3 weeks until the end of the co-incubation. β-1,3-Glucanase activity was also detected on day 7 and the same level of the activity was maintained for 3 weeks. Strains TW10 and TB10, when compared to one another, showed a similar pattern of changes in the products as well as the activity levels. The amount of butyrate produced by the two strains was approximately double as that of acetate throughout the co-incubation. Although the β-1,3-glucanase activity levels were approximately half of that of strain TW1, the levels were also maintained for 3 weeks.

### Observation of mycelial cells of the *Fusarium* spinach wilt pathogen co-incubated with the anaerobic bacterial strains by fluorescence microscopy

**Fig. 4:**
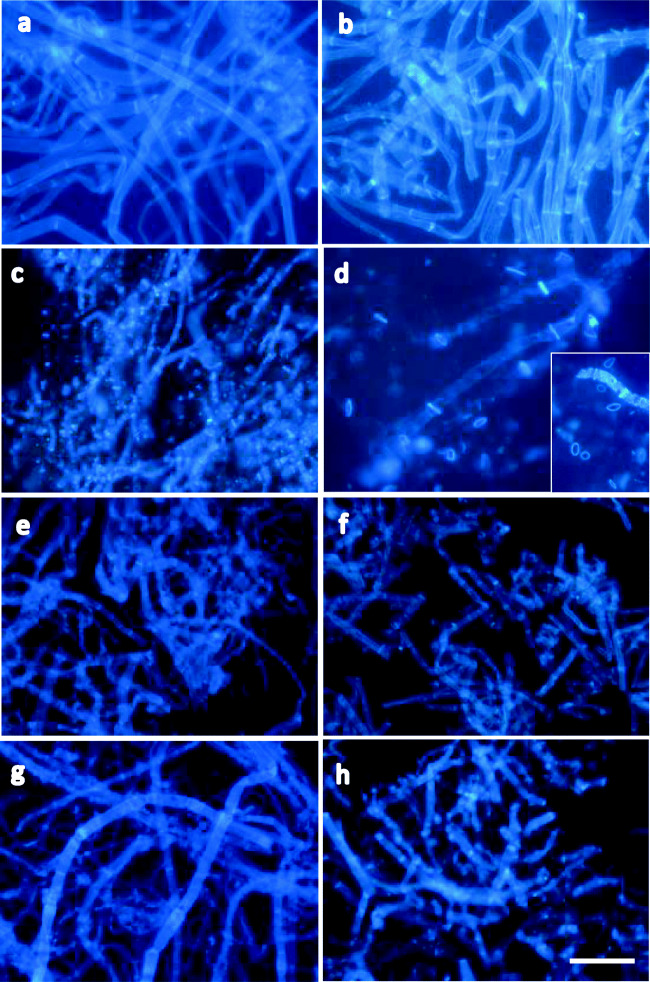
Fluorescence photomicrographs of cells of *Fusarium oxysporum* f. sp. *spinaciae* strain M2-1 anaerobically incubated alone (control) or anaerobically co-incubated with strains TW1, TW10, and TB10 in PY broth containing wheat bran (1%, w/v) at 30 °C. Living mycelial cells of strain M2-1 on agar plate sections of PDA were inoculated to the medium under the stream of O_2_-free gas, and the agar sections harvested after each incubation period were used for the microscopic observation. Control (**a** day 0; **b** day 21). Co-incubation with TW1(**c** day 7; **d** the insert; day 21 of different microscope fields); TW10 (**e** day 7; **f** day 21); TB10 (**g** day 7; **h** day 21). Bar, 30 μm

The mycelial cells of strain M2-1 on agar pieces that were co-incubated anaerobically with each bacterial strain in the presence of wheat bran were observed by fluorescence microscopy using calcofluor white (Fig. 4). Whole cells of strain M2-1 at the start of co-incubation emitted bright fluorescence with strong and clear emission at the septa of mycelia (Fig. 4a). The cells anaerobically incubated alone in PY broth containing wheat bran for 3 weeks showed nearly the same fluorescence as did the start of co-incubation, which indicates that the anaerobic incubation under the presence of wheat bran itself did not result in any evident effects on the cell wall of the pathogen (Fig. 4b). Mycelial cells co-incubated with strain TW1 for 7 days were apparently damaged and the cell shape became obscure, whereas the septa with strong fluorescence were scattered in the microscope field as dots or short bars (Fig. 4c). After the co-incubation for 3 weeks with strain TW1, the mycelial cells were nearly destroyed and the septa as luminous clear rings or bars remained, which reveals the persistence of the septa to the enzyme activity (Fig. 4d).

When compared to the intense effects of strain TW1 on the pathogen cells, the effects of strains TW10 and TB10 on the mycelia seemed not to be so severe (Fig. 4e–h). Mycelial cells co-incubated with strains TW10 or TB10 maintained the cell form until day 7 of incubation, though the cell wall of the pathogen seemed to become unevenly thin with transparent patches (Fig. 4e, g). Co-incubation for 3 weeks with strain TW10 or TB10 did not seem to add further notable damages on the pathogen cells (Fig. 4f, h), although many of the mycelial cells appeared to be cut at the septa emitted bright fluorescence.

### Decomposition of dead whole cells or cell wall of the *Fusarium* pathogen by the anaerobic strains

To confirm the ability of the three bacterial strains to utilize or decompose whole cell biomass or the cell wall of *F. oxysporum* f. sp. *spinaciae* as direct growth substrates, the three strains were cultivated with these samples that were prepared from the *Fusarium* pathogen strain M2-1. Because the PY medium was autoclaved after adding the freeze-dried fungal biomass to the medium, the fungal cells were not alive. Table 5 shows the amounts of products and the enzyme activities in the supernatants that were cultivated with these substrates.

**Table 5:** End products and β-1,3-glucanase activity of strains TW1, TW10, and TB10 in the culture supernatants grown with the cell biomass or cell wall preparations of the *Fusarium* pathogen of spinach wilt disease

Strain	Substrate	Days*	Products (mmol/l)		β-1,3-Glucanase (unit/ml)
			Acetate	Butyrate	
TW1	*Fusarium* biomass	7	8.11 ± 0.79	7.50 ± 0.41	3.94 ± 0.39
	*Fusarium* cell wall	7	10.1 ± 0.21	11.1 ± 0.52	4.86 ± 0.78
	*Fusarium* cell wall	14	10.4 ± 0.27	10.8 ± 0.04	5.50 ± 1.00
	*Fusarium* cell wall	21	10.3 ± 0.41	10.5 ± 0.13	4.89 ± 0.62
TW10	*Fusarium* biomass	7	1.70 ± 0.03	2.70 ± 0.06	0.24 ± 0.02
	*Fusarium* cell wall	21	2.27 ± 0.03	6.00 ± 0.01	0.38 ± 0.04
TB10	*Fusarium* biomass	7	1.70 ± 0.08	3.00 ± 0.02	0.09 ± 0.03
	*Fusarium* cell wall	21	1.63 ± 0.12	6.23 ± 0.25	0.71 ± 0.08

*Fusarium* biomass, freeze-dried mycelial cells of *Fusarium oxysporum* f. sp. *spinaciae* strain M2-1; *Fusarium* cell wall, a cell wall preparation of strain M2-1

Concentrations of substrates, *Fusarium* biomass, 1% (*w*/*v*); *Fusarium* cell wall, 0.5% (*w*/*v*)

Products, mean ± SD (*n* = 2); β-1,3-glucanase, mean ± SD (*n* = 4)

*Days of cultivation

Strain TW1 produced similar product amounts from the fugal whole cell biomass as did those from wheat bran (Table 4), which suggests that the fungal biomass directly supported strain growth and served as a substrate. In accordance with the amounts of products, a high activity of β-1,3-glucanase was detected from the culture supernatant. Moreover, strain TW1 produced higher amounts of acetate and butyrate from the *Fusarium* cell wall sample compared to the wheat bran or dead cell biomass samples until day 7 of incubation, thus indicating that strain TW1 indeed had the ability to easily and directly decompose and use ascomycetes cell wall as a growth substrate. When strain TW1 was further cultivated with the cell wall sample as a growth substrate, nearly the same amounts of products and β-1,3-glucanase activities were detected from the cultures on days 14 and 21 (Table 5).

When strains TW10 and TB10 were cultivated for 7 days with the dead *Fusarium* whole cells, both strains did not show increases in the product amounts compared to the amounts from the PY broth (Table 2), and only low enzyme activities were detected (Table 5). The products and the activities for strains TW10 and TB10 with the cell wall preparation were determined for the culture supernatants that had been cultivated for 3 weeks. The detected β-1,3-glucanase activities were low with the similar levels to those obtained from the PY broth for both strains, although the butyrate amounts produced were slightly higher than those produced with the PY broth (Table 2). These results show that strains TW10 and TB10 were not effective in decomposing the dead whole cells or the cell wall of the *Fusarium* pathogen as growth substrates, and did not produce β-1,3-glucanase by depending on these materials.

### Observation of the mycelial whole cells and the cell wall preparation of the *Fusarium* pathogen incubated with the anaerobic strains by fluorescence microscopy

The mycelial cells of the dead biomass and the cell wall preparation of strain M2-1—after incubation with each anaerobic strain—were observed by fluorescence microscopy with calcofluor white stain. Supplemental Fig. S2a shows the fluorescence photomicrograph of the dead whole cells at the start of incubation. The entire mycelial cells had emitted bright fluorescence, as such the septa are clearly portrayed with the stronger luminous intensity, which is a manner similar to that for living cells shown above in Fig. 4a. Nearly identical fluorescence images were observed for the whole cells that were incubated anaerobically alone for 7 days in PY broth (data not shown). Supplemental Fig. S2b–d shows the fluorescence photomicrographs of the *Fusarium* cells that was incubated with each anaerobic strain for 7 days. As for strain TW1 (Supplemental Fig. S2b), the *Fusarium* cells were severely destroyed and the cell outlines were obscure with highly luminous septa or septum rings remaining. In contrast, for the *Fusarium* cells incubated with either strain TB10 (Supplemental Fig. S2c) or TW10 (Supplemental Fig. S2d), the apparent effects on the cells were not observed.

**Fig. 5:**
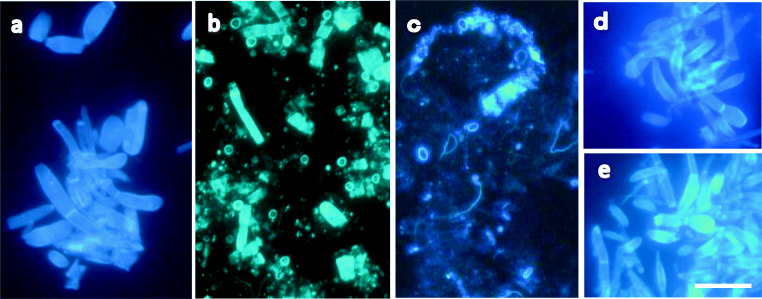
Fluorescence photomicrographs of the cell wall samples of *Fusarium oxysporum* f. sp. *spinaciae* strain M2-1 incubated under the anaerobic conditions at 30 °C. Cell wall preparation **a** at the start of the incubation, incubated with **b** strain TW1 (day 3), **c** strain TW1 (day 7), **d** strain TW10 (day 21), and **d** strain TB10 (day 21). Fluorescence emitted from the cell wall debris incubated with strain TW1 for 14 or 21 days was too weak to confirm the cell shapes (data not shown). Bar, 60 μm

Figure 5a shows a fluorescence photomicrograph of the cell wall preparation of strain M2-1 at the start of incubation with the anaerobic strains. The cell wall preparation was mainly composed of disrupted ghost cells that were cut off at the septa. The cell wall sample incubated with strain TW1 for 3 days was already nearly degraded, and the luminous septum rings were clearly observed (Fig. 5b). The cell wall preparation—incubated for 7 days with strain TW1—was almost thoroughly degraded. Figure 5c shows the ring-like septa that remained, but it was mostly difficult to confirm the shape or outline of the cells even by the careful microscopic observation. After 2 or 3 weeks of incubation, the cell shape was hardly confirmed using only the obscure emission from the cell debris. In contrast, the cell wall preparation—incubated with strains TW10 (Fig. 5d) or TB10 (Fig. 5e)—did not show any deterioration even after 3 weeks of incubation.

## Discussion

Based on the analysis of 16S rRNA gene sequences, most of the 20 anaerobic bacterial isolates from the soil samples that were subjected to BSD treatments were affiliated with the genus *Clostridium* in the class *Clostridia* (Lawson and Rainey 2016). Although most strains were saccharolytic and had produced similar fermentation end products, the abilities to decompose β-1,3-glucan or chitosan were restricted to the four groups in the isolates. The strains that were shown to decompose both β-1,3-glucan and chitosan were assigned to a single lineage that was identified as *C. beijerinckii*, whereas each of the three strains that decomposed β-1,3-glucan and not chitosan was classified into different groups of the genus *Clostridium*. Although certain strains, such as strains TW8 and H18, were closely related to the β-1,3-glucan- or chitosan-degrading strains based on the 16S rRNA gene sequences (Fig. 1), none of them decomposed the respective polysaccharides (Table 2). Therefore, although most clostridial isolates showed similar physiological properties—especially in fermentation of saccharoses to similar end products—they may each have their own role in accordance with their specific substrates that are environmentally available. The 16S rRNA gene sequence similarity (96.1%) of strain TW1, with the closest relative *C. polyendosporum*, suggests that the strain represents a novel species in the genus *Clostridium*. Thus, further polyphasic characterization of the strain is required to confirm its taxonomic assignment. The closest species of strain TW10, *C. saccharoperbutylacetonicum*, utilizes various carbohydrates and produces ethanol, butanol, and acetone, in addition to acetate, butyrate, H_2_, and CO_2_ (Rainey et al. 2009). The saccharolytic features of strain TW10 matched the description of *C. saccharoperbutylacetonicum*. However, strain TW10 did not produce solvents from examined carbohydrates. Thus, although the 16S rRNA gene sequence similarity of strain TW10 with *C. saccharoperbutylacetonicum* was high (99.1%) and strain TW10 had similar physiological properties with *C. saccharoperbutylacetonicum* for utilization of various carbohydrates (Supplemental Table S2), we currently were not able to confirm the identification of the strain as *C. saccharoperbutylacetonicum*. The 16S rRNA gene sequence similarity of strain TB10 with its closest relative *C. chromiireducens* was 99.2%, and the cellular and physiological characteristics of the strain (Supplemental Table S2) coincided considerably with the characteristics of *C. chromiireducens* (Inglett et al. 2011). Therefore, based on these data, strain TB10 should be identified as *C. chromiireducens*.

The bacterial composition of the BSD-treated soil samples that were subjected to isolation of the anaerobic strains examined in this study had been analyzed by clone library analyses in our previous studies (Mowlick et al. 2012, 2013a). The sequence similarities of strains TW1, TW10, and TB10 with their respective closest clones ranged from 98.5 to 99.8% (Fig. 1, Supplemental Table S1). Although the sequence similarity of strain TW1 with its closest described species was 96.1%, as shown above, the similarity with the closest clone was 99.8%. These similarity values suggest that each strain represents a closely related bacterial group that was in fact proliferated in each of the BSD-treated soil samples.

β-1,3-Glucan, β-1,6-glucan, chitin, and chitosan are major polysaccharides composed of the cell wall of ascomycetes fungi. Among these polysaccharides, β-1,3-glucan is the most abundant at 70 to 80% and β-1,6-glucan and chitin each comprise less than 10% (Arroyo et al. 2016; Geoghegan et al. 2017; Ruiz-Herrera and Ortiz-Castellanos 2010; Schoffelmeer et al. 1999). Some aerobic bacteria produce β-1,3-glucanase and chitosanase that exhibit anti-fungal activities (Aktuganov et al. 2007; Kurakake et al. 2013; Prasanna et al. 2008). We reported the degradation of fungal cell wall and production of anti-fungal enzymes (β-1,3-glucanase and chitosanase) by the obligate anaerobic bacterial strains (H110 and TB8) of *C. beijerinckii* that were isolated from the BSD-treated soil samples (Ueki et al. 2017, 2019). The production of β-1,3-glucanase by an obligate anaerobic bacterium *Hungateiclostridium thermocellum* (formerly *Clostridium thermocellum*) (Zhang et al. 2018) has also been previously reported (Dvortsov et al. 2009).

Curdlan and laminarin supported active growth of the three strains (TW1, TW10, and TB10), and production of β-1,3-glucanase was confirmed for all the three strains in the culture supernatants with these glucans. Wheat bran was also found to be an excellent substrate for growth and β-1,3-glucanase production in regard to these strains. Starch and non-starch polysaccharides, including cellulose, lignin, and xylan (mainly composed of arabinose and xylose), are major components of wheat bran, along with a small amount (2–3% *w*/*w*) of β-glucan (Chalamacharla et al. 2018; Kabel et al. 2002; Maes and Delcour 2002). Although it is uncertain what types of compounds in wheat bran are effective in inducing β-1,3-glucanase production of these strains, wheat bran was confirmed to have a potential to stimulate both growth and β-1,3-glucanase production of the anaerobic bacteria. In our previous studies, we have also reported that wheat bran supports growth and production of enzymes (both β-1,3-glucanase and chitosanase) of strains H110 and TB8 (Ueki et al. 2019). Thus, wheat bran has further been proven a suitable substrate for stimulating growth of anaerobic bacteria with the ability to produce anti-fungal enzymes that suppress soil-borne fungal plant pathogens. *Brassica* plants also had the potential to support growth and β-1,3-glucanase production of strain TW1, whereas strains TW10 and TB10 utilized it only to a weak degree for growth and the enzyme production.

The time course study of the anaerobic co-incubation in the presence of wheat bran showed that the three bacterial strains grew rapidly in the initial period of the co-incubation and the concentrations of products and the β-1,3-glucanase activity did not change after day 7 of the incubation (Fig. 3). BSD treatments in the field soil are typically carried out for approximately 3 weeks. Therefore, we also conducted the co-incubation experiment for 3 weeks to know the effects of the enzymes (or other products) formed by the anaerobic bacteria on the *Fusarium* pathogen throughout the whole period of BSD treatments. The living cells of the *Fusarium* pathogen that were anaerobically co-incubated with each anaerobic strain were no longer viable by day 7 of the co-incubation. Furthermore, the enzyme β-1,3-glucanase that was produced in accordance with the growth of each strain until day 7 degraded the pathogen cells more severely or thoroughly during the prolonged anaerobic co-incubation for 3 weeks. The results showed that β-1,3-glucanase, once it is produced by the bacteria in soil, has the potential to maintain the activities and thus to give damage to soil-borne fungal pathogens for the entire period of the treatments—under the conditions that the enzyme proteins were not decomposed by other microbes that live in the soil. β-1,3-Glucanase and chitosanase, which were produced by strains H110 and TB8, were also stable during the anaerobic incubation period that lasted 3 weeks (Ueki et al. 2019).

Calcofluor white has often been used to stain chitin in fungal cell wall to observe it by fluorescence microscopy. The *Fusarium* pathogen strain M2-1 that had been used as the representative soil-borne plant pathogen in this study is an ascomycetes fungus, which has chitin as a cell wall component. In this study, fluorescence microscopy revealed that the mycelial cells of the living *Fusarium* pathogen co-incubated in wheat bran with each anaerobic bacterial strain were attacked in different manners that depend on the bacterial strains. Further, strain TW1 was shown to significantly degrade the mycelial sidewall in the early period of the co-incubation, although the mycelial septa remained unique rings or bars that emit strong fluorescence. In contrast, for co-incubation with strains TW10 and TB10, most pathogen cells were found to maintain their shape, even after the co-incubation period of 3 weeks, although the mycelial sidewall of the pathogen seemed to become unevenly fragile with translucent patches. Strains H110 and TB8, which are the producers of both β-1,3-glucanase and chitosanase, uniformly degraded the fungal cells irrespective of the parts of mycelia (sidewall or septa) and thoroughly and quickly destroyed the cells in the short duration of the co-incubation period (Ueki et al. 2019). Chitin is generally rich in the inner layer of the cell wall of most fungi (Munro and Gow 2001) and is also a major component of the septa in fungal mycelia (Horiuchi 2009; Hunsley and Gooday 1974; Mouriño-Pérez 2013). Chitosan in fungal cell wall is synthesized by the deacetylation of GlcNAc in the chitin chains (Geoghegan and Gurr 2017; Ruiz-Herrera and Ortiz-Castellanos 2010). Further, it is reported that approximately 65 to 75% of the chitinous component of *F. oxysporum* is deacetylated (Fukamizo et al. 1996). Therefore, we speculated in the previous study that strains H110 and TB8 attacked the deacetylated chains in the cell wall chitin of the *Fusarium* pathogen using chitosanase and proceeded to degrade the entire cell wall, including the chitin-rich septa, using the synergistic activities with β-1,3-glucanase and chitosanase (Ueki et al. 2019). The three strains of the present study did not decompose chitosan, and in fact, chitosanase activity for all strains was not detected in the supernatants of the culture with wheat bran (data not shown). The results obtained in this study suggest that although β-1,3-glucanase can act alone as a fully effective anti-fungal agent, coexistence of chitosanase seems to be a key to thoroughly degrade fungal cells including the septa.

Strain TW1 was shown to easily decompose both the dead *Fusarium* whole cells and the cell wall sample as direct substrates. Based on the product amounts in the culture supernatant of strain TW1, the cell wall preparation appeared to be a favorable substrate comparable to curdlan, and an even better substrate than the commercially available yeast glucan. In contrast, strains TW10 and TB10 barely decomposed the *Fusarium* whole cells and the cell wall sample. Thus, although β-1,3-glucanase is commonly produced by strains TW1, TW10, and TB10, and these strains kill the living *Fusarium* pathogen under the anaerobic co-incubation in the presence of wheat bran, the mechanisms to killing the pathogen or the enzymatic properties of the produced β-1,3-glucanase are different depending on the strains. Strain TW1 is able to produce the enzyme using the fungal biomass or cell wall itself as direct growth substrates, in addition to other substrates, such as wheat bran, and ultimately decomposes the cell wall (or cell wall glucans) of the fungal pathogen almost completely, except for the mycelial septa. In contrast, production of β-1,3-glucanase for strains TW10 and TB10 seems to be entirely dependent on the presence of appropriate growth substrates, such as the glucans or wheat bran. The complex structure of the fungal cell wall as a solid material may prevent induction of β-1,3-glucanase production of the two strains. Strains H110 and TB8 also easily decomposed both the fungal whole cells and the cell wall sample, as growth substrates, and the fungal biomass supported the production of β-1,3-glucanase and chitosanase of these strains (Ueki et al. 2019). Therefore, strain TW1 is alike to strains H110 and TB8 in that it has the advantage of directly attacking the fungal cells irrespective of whether it is alive and easily degrades the solid fungal biomass.

We previously reported the production of the anti-fungal enzymes (β-1,3-glucanase and chitosanase) of strains H110 and TB8 was also derived from the BSD-treated soil samples (Ueki et al. 2017, 2019). The results obtained in this study further confirm the presence of diverse clostridial groups that produce β-1,3-glucanase and result in fatal damages on the ascomycetes fungal pathogens in the BSD-treated soil. It was also shown that the mechanisms to degrade fungal cells were different for each bacterial group. These anti-fungal enzymes that are produced extracellularly by various anaerobic bacterial groups certainly play key roles in the elimination of soil-borne ascomycete pathogens using different mechanisms that attack fungal cells. It is probable that other enzymes that have similar abilities to inactivate soil-borne pathogens are also produced by other soil-grown bacterial groups and act cooperatively as anti-pathogenic agents. As shown in Table 2, most of the anaerobic strains in our study isolated from the BSD-treated soil samples utilized GlcNAc and glucosamine as growth substrates. Therefore, these monomers, including glucose, released by the activities of polysaccharide-decomposing bacterial groups may support growth of other many bacterial groups without the abilities to degrade the polysaccharides in soil under the BSD treatments. Characteristics and kinetics of the produced β-1,3-glucanases by the anaerobic strains from the BSD-treated soil samples, including H110 and TB8, should be further examined to clarify the differences in the cell wall degradation mechanisms of ascomycete fungi. Gene sequences coding theses enzymes should also be analyzed to better understand the phylogenetic relationships among the enzymes.

## Electronic supplementary material

ESM 1 (PDF 8735 kb)
