# Supplementary material for: β-1,3-Glucanase production as an anti-fungal enzyme by phylogenetically different strains of the genus Clostridium isolated from anoxic soil that underwent biological disinfestation
Source: Appl Microbiol Biotechnol. 2020 Apr 24;104(12):5563–78. doi: 10.1007/s00253-020-10626-8 (PMC7275012; doi:10.1007/s00253-020-10626-8)
Supplement: Supplementary file 1 — (PDF 8735 kb) [file 253_2020_10626_MOESM1_ESM.pdf]

Applied Microbiology and Biotechnology

Supplementary materials

β-1,3-Glucanase production as an anti-fungal enzyme by phylogenetically different strains of the genus *Clostridium* isolated from anoxic soil that underwent biological disinfestation

Atsuko Ueki[a*], Toshiaki Takehara[b**], Gen Ishioka[b], Nobuo Kaku[a], and Katsuji Ueki[a]

[a]Faculty of Agriculture, Yamagata University, Yamagata 997-8555, Japan

[b]NARO Western Region Agricultural Research Center, Hiroshima 721-8514, Japan

[*]**Corresponding author:** Dr. Atsuko Ueki

Yamagata University, 1-23, Wakaba-machi, Tsuruoka Yamagata 997-8555, Japan. Tel: +81 235-28-2846; Fax: +81 235-28-2846

E-mail: uatsuko@tds1.tr.yamagata-u.ac.jp

[**]Present address: Toshiaki Takehara

NARO Technical Support Center of Central Region, Ibaraki 305-8517, Japan

Table S1. 16S rRNA gene sequence similarities of strains TW1, TW10, and TB10 with the closest known species and the representative closest clone obtained from the same soil sample

Strain	Accession No.	Closest species			Closest clone from the same soil sample		
		Species and the type strain	Accession No.	Similarity (%)	Clone	Accession No.	Similarity (%)
TW1 (= NBRC 112097)	LC020506	*Clostridium polyendosporum* DSM 5272^T	Y18189	96.1	WB 9-38	AB589916	99.8
TW10 (= NBRC 112099)	LC020495	*Clostridium saccharoperbutylacetonicum* N1-4(HMT)^T	U16122	99.1	WB 9-66	AB589936	99.3
TB10 (= NBRC 112095)	LC020496	*Clostridium chromiireducens* GCAF-1^T	AY228334	99.2	BR 9-13	AB589738	98.5

Accession No., 16S rRNA gene sequence accession numbers of DDJB.

Table S2. Some characteristics of strains TW1, TW10, and TB10

Characteristics	TW1	TW10	TB10
Gram stain	+	+	+
Cell morphology	Slender rods	Short rods	Short rods
Spore	+	+	+
Motility	-	+	+
Catalase	-	-	-
Substrate utilization			
Arabinose	+	+	-
Cellobiose	+	+	+
Glucose	+	+	+
Lactose	+	+	+
Maltose	+	+	+
Mannose	+	+	+
Melezitose	+	+	-
Raffinose	+	+	+
Rhamnose	+	+	+
Sucrose	+	+	+
Trehalose	+	+	+
Xylose	+	+	+
Esculin	-	+	+
Salicin	+	+	+
Glycerol	+	+	-
Mannitol	+	+	+
Sorbitol	+	+	-
Gelatin hydrolysis	-	+	-
Urease production	-	-	-
Indole production	-	-	-
H_2S production	+	-	-

Carbohydrate utilization, gelatin hydrolysis, and urease production were determined with API 20A system (bioMérieux) in duplicate according to the instructions of the manufacturer. Production of indole and H_2S was determined using SIM medium.

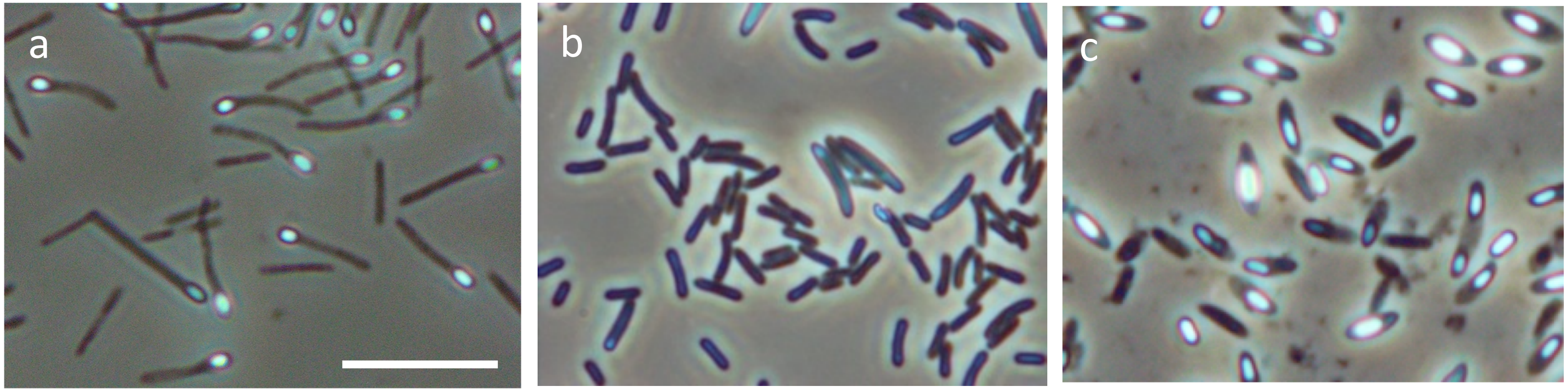

Fig. S1. Phase-contrast photomicrographs of cells of strains (a) TW1, (b) TW10, and (c) TB10 grown in PYG broth. Bar, 10 μm.

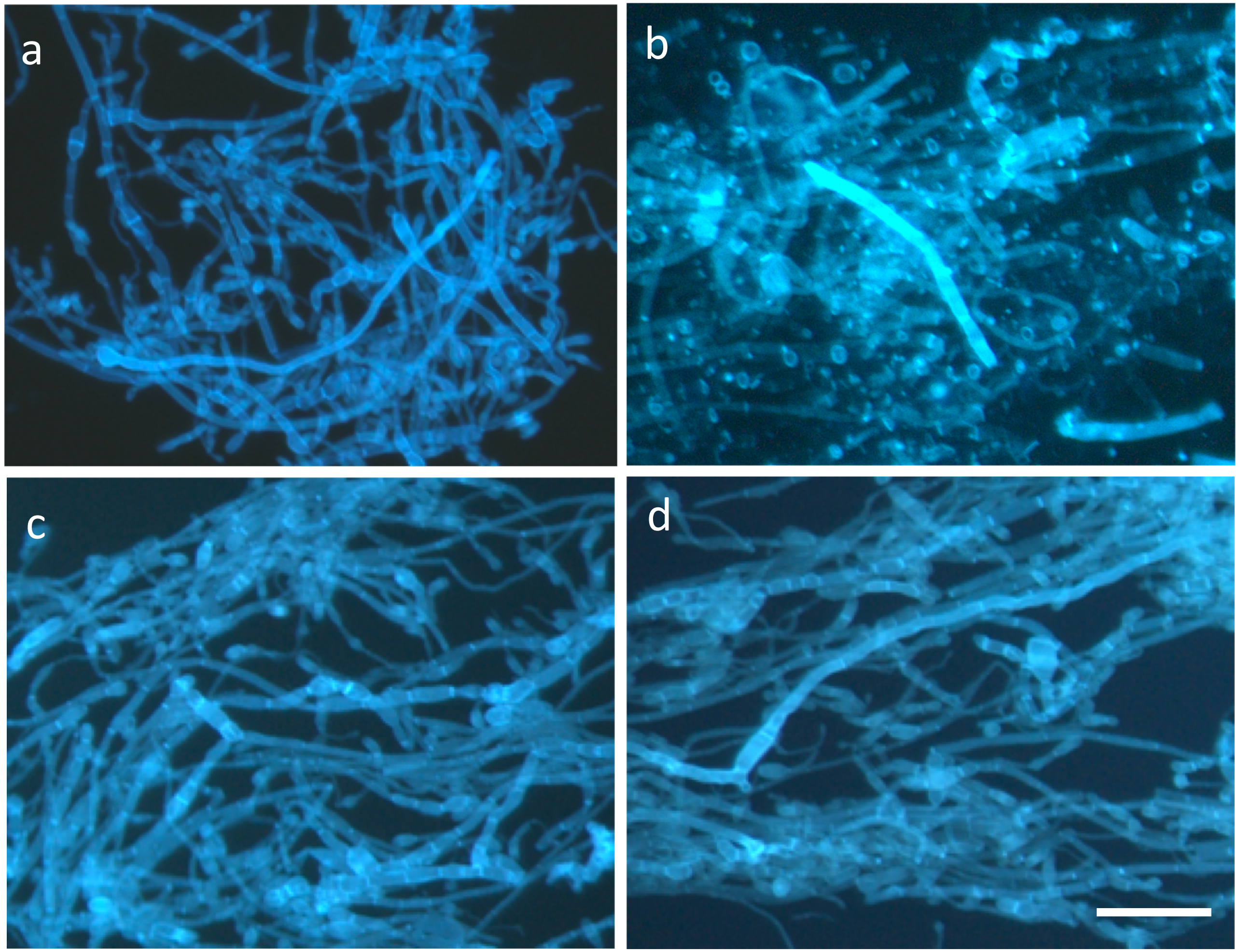

Fig. S2. Fluorescence photomicrographs of cells of *Fusarium oxysporum* f. sp. *spinaciae* strain M2-1 (a) before incubation or anaerobically incubated at 30°C for 7 days with strains (b) TW1, (c) TW10 or (d) TB10 in PY broth containing the dead *Fusarium* cells (1%, w/v). Bar, 30 µm.
